# The effect of desacetyluvaricin on the expression of TLR4 and P53 protein in Hepg 2.2.15

**Published:** 2011-05-01

**Authors:** Hai Bin He, Xian Lin Wu, Bin Yu, Kang Li Liu, Guang Xiong Zhou, Guo Qiang Qian, Da Hong Ju, Xiao Yin Chen

**Affiliations:** 1 Department of Gastroenterology, the People’s Hospital of Heshan, Guangdong, China; 2 Department of Traditional Chinese Medicine, Medicine College of Jinan University, Guangzhou, China; 3 Pharmaceutical College of Jinan University, Guangzhou, China; 4 Institute of Basic Theories of Traditional Chinese Medicine, China Academy of Traditional Chinese Medicine, Beijing, China

**Keywords:** Desacetyluvaricin, TLR4 receptor, P53 tumor suppressor protein, Flow cytometry, Hepatocellular carcinoma

## Abstract

**Background:**

Previous studies suggest that annonaceous may cause permeability glycoprotein (P-gp) function to abate, leading to cell apoptosis. It has also been reported that annonaceous acetogenins affect hepatocellular carcinoma (HCC) cells in the G1 phase, leading to apoptosis. Desacetyluvaricin (Des), a new type of annonaceous acetogenin monomer, has a significant effect on HCC, with few side effects.

**Objectives:**

To investigate the effect of Des on the expression of Toll-like receptor 4 (TLR4) and P53 protein in HCC.

**Materials and Methods:**

HCC HepG2.2.15 cell was cultured by routine method. HepG2.2.15 cells were divided into three groups: control group, treated with Des and DDP (cisplatin) which were examined by immunofluorescence flow cytometry for expression of TLR4 and P53.

**Results:**

TLR4 was expressed by more cells in the Des group than in the cisplatin or serum-only groups (71.94%, 42.64%, and 37.16%, respectively; Des vs.cisplatin: p < 0.05; Des vs. serum only: p < 0.05), with no difference between the cisplatin and serum-only groups (p > 0.05). P53 was expressed by more cells in the Des and cisplatin groups than in the serum-only group (32.6%, 31.5% and 3.3%, respectively; Des vs. serum only, p < 0.05; cisplatin vs. serum only, p < 0.05), with no difference between the Des and cisplatin groups (p > 0.05).

**Conclusions:**

Des increases TLR4 and P53 expression in HCC cells. Improved immune recognition by the former effect and induction of apoptosis by the latter could be the mechanisms of Des's clinical effects on HCC.

## Background

In 1982, Tempesta isolated uvaricin, a lactone with strong anti-cancer activity, from the roots of Uvaria accuminata of the family Annonaceae [1]. Annonaceous acetogenin compounds are considered a promising natural source of anti-tumor compounds, and an increasing amount of research is being done on this compound. Studies suggest that annonaceous acetogenins' anti-tumor mechanism may be through inhibition of mitochondrial NADH oxido-reductase, preventing the transmission of respiratory chain electrons and producing a rapid decrease in ATP levels. This, in turn, causes a decrease in permeability glycoprotein (P-gp) function, leading to cell apoptosis [2]. It is reported that annonaceous acetogenins affect tumor cells in the G1 phase, inducing BAX expression, increasing caspase-3 activity, and finally inducing apoptosis [3][4]. However, it is not clear whether annonaceous acetogenin's anti-tumor effect is achieved by increasing immune function.

Des (Des), a type of annonaceous acetogenin monomer extract from the traditional Chinese medicine, has demonstrated a significant effect on liver cancer [5]. Our research group previously showed that Des can block mitosis in hepatocellular carcinoma (HCC) cells in vitro, inhibiting cell proliferation [6]. We found that Des induced Fas expression better than cisplatin (Cis-diammin-odichloroplatinum Ц dichloridle, DDP). We also showed that Des's effect on inhibiting tumor cells was similar to chemotherapy drug cisplatin. However, when hepatitis B virus (HBV) was added to the cell cultures, Des's effect to induce Fas expression was greater than chemotherapy drug cisplatin. We speculated that Des may enhance immune function.

## Objectives

To further elucidate the anti-HCC role of Des and its mechanism in enhancing immune function on the HepG2.2.15, we used monomer and DDP to intervene, and then detect the expression of TLR4 and P53, to observe the effects on HepG2.2.15 treated by Des and hope to reveal the mechanism.

## Materials and Methods

### Reagents and instruments

Dulbecco's modified Eagle medium (DMEM) was produced by GIBCO Company, USA; fetal calf serum (FCS) was produced by SiJiQing Biomedical Materials Engineering Research Institute, Hangzhou, China; Phycoerythrin (PE)-TLR4 antibody (PE-anti-TLR4) and PE-mouse IgG2a was produced by Ebioscience Company, San Diego, USA; PE-anti-P53 was produced by AbDSerotec Company; BD FACS Calibur flow cytometer was produced by COULPER Company and provided by the First Affiliated Hospital of Sun Yat-sen University.

### Treatments

**Figure 1:**
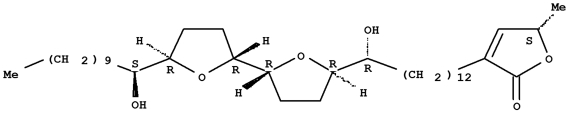
Des chemical structure (molecular formula C37H66O6)

DDP was provided by Qilu Pharmaceutical CO, Ltd, Jinan, China, batch number: 703012CF; Des was provided by Guang Xiong Zhou who produced it in his laboratory, Pharmaceutical College of Jinan University. (Figure 1).

### Cancer cell line

The HCC cell line HepG2.2.15 was provided by Ming-xia Zhang, Southern Medical University in Guangzhou, China.

### Cell culture and treatment exposure

HepG2.2.15 cells were incubated in 10% inactivated FCS DMEM (pus G418) at 37°C and 5% CO2 in normal culture passage. Logarithmic phase cells were selected with 1 mL of 0.25% trypsin, washed, replated onto a 12-well plate at a concentration of 3×104 cells/mL, and incubated at 37°C and 5% CO2 for 48 hours. At the 48th hour, the cells entered the exponential growth phase. Cells were then washed and 0.5 mL of either FCS (negative control), FCS containing 100 µg/mL Des, or FCS containing 10 ?g/mL DDP (positive control) was added to relevant wells [7]. The culture plate was then incubated for another 48 hours at 37°C and 5% CO2. Five such plates were prepared.

### Detection of TLR4 and P53 expression

After incubation, cells were washed and tagging with fluorescent antibodies for either TLR4 or P53, as follows [8][9]. For each of the three treatment groups, cells were incubated for 48 hours, 1 mL of 0.25% trypsin was added to digest, then centrifuged to collect cells, and then combined into a single suspension of 1×106 cells. For P53 measurements, cells were incubated instead with 1% Triton X-100; then we added 10 μL PE-anti-P53 and 100 μL cells suspension. For TLR4 measurements, we added 10 μL PE-anti-TLR4 and 100 μL cells suspension. Cells were washed with phosphate buffered saline (PBS). We followed the above-mentioned methods to make the same type of control except that we used 10 μL of PE-mouse IgG2a instead of PE-anti-TLR4 or PE-anti-P53, as control group. The suspensions were then mixed and incubated for 20 minutes at room temperature in the dark; then suspensions of all the studied groups were centrifuged at 1000 rps for 5 minutes; PBS was added to 106/mL dubbed liquid. The cells were examined by direct immunofluorescence flow cytometry [10] and the number of positive cells was counted. Five such plates were prepared.

### Statistical analysis

The results were presented as Mean ± SD. SPSS® for Windows® (Ver 13.0; SPSS, Chicago, IL, USA) was used for data analysis. The proportion of positive cells was calculated for each of the five experiments and the results were then averaged. The treatment and control groups were evaluated using analysis of variance. A p < 0.05 was considered statistically significant.

## Results

### Expression of TLR4 after Des

**Figure 2:**
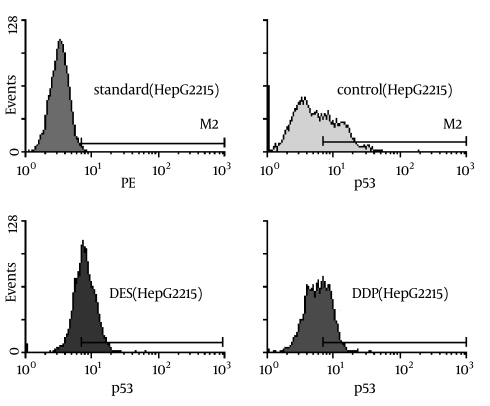
TLR4 expression in HepG2.2.15, measured by immunofluorescence flow cytometry. X-axis is the cell number, Y-axis is the fluorescence. CD284: TLR4expression; Standard: Standard calibration. Control: serum-only treated; DES: Desacetyluvaricin-treated; DDP: Cisplatin-treated

Among Des-treated cells, the Mean ± SD TLR4-positive rate was 71.94% ± 1.04%, significantly higher than that observed in the DDP group (42.64% ± 0.83%, p < 0.001) or the serum-only group (37.16% ± 1.33%, p < 0.001) (Figure 2 and Table 1). There was no significant difference between the DDP and serum-only groups (p = 0.128).

**Table 1:** Mean ± SD expression of TLR4 (CD284) and P53 (No. = 5)

**Group**	**Positive rate of CD284** (%)	**Positive rate of P53** (%)
**Control**	37.16 ± 1.33	3.3 ± 0.6
**Desacetyluvaricin**	71.94 ± 1.04 a,b	32.6 ± 1.7^a^,^b^
**DDP**	42.64 ± 0.83 a	31.5 ± 1.3%

^a^ Significantly (p < 0.05) different from control group

^b^ Significantly (p < 0.05) different from DDP group

### Expression of P53 after Des

**Figure 3:**
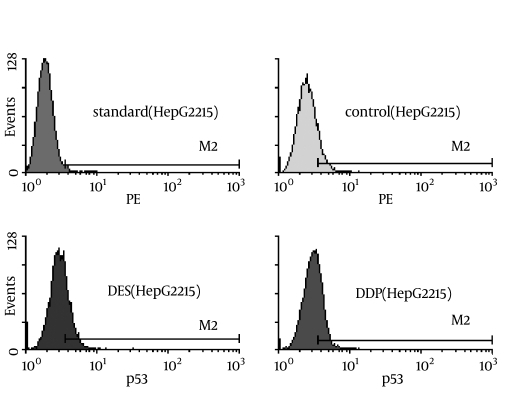
P53 expression in HepG2.2.15 hepatocellular carcinoma cells, measured by immunofluorescence flow cytometry. Standard: Standard calibration. Control: Serum-only treated; DES: Desacetyluvaricin-treated; DDP: Cisplatin-treated

The Mean ± SD P53-positive rates in the Des, DDP and serum-only groups were 32.6% ± 1.7%, 31.5% ± 1.3%, and 3.3% ± 0.6%, respectively (Figure 3 and Table 1). There was no difference between the Des and DDP groups (p = 0.000).The expression of P53 in both groups was significantlyhigher than that in the serum-only group (p < 0.001 for both).

## Discussion

TLRs play an important role in the host immune response to pathogenic microorganisms. They can identify molecules of pathogenic microorganisms in the innate immune system, and initiate the innate immune response. TLRs also can induce cytokine release, increase the expression of co-stimulatory molecules, and initiate the acquired immune response [11]. Recent research has shown that TLR4-deficient mice have a higher incidence of lung cancer than normal mice, suggesting that TLR4 has a preventive role in genesis of tumors [12]. We found that Des increased expression of TLR4 in HCC cells. This mechanism, via TLR4's stimulation of the immune response, may explain Des' inhibiting effect on HCC.

P53, which is encoded by the tumor suppressor gene P53, has two independent HBV X protein (HBx) binding sites, located in the P53-specific DNA binding and oligomerization areas [13]. HBx-P53 complexes have been found in the cytoplasm of liver cells; it has been shown that their binding is reversible. By binding to P53, HBx prevents P53 trans-activation, thus inhibiting P53-guided apoptosis [14]. This may play an important role in HBV's hepatocarcinogenesis. In liver nuclei, low levels of P53 support cell division at the G1 phase and block apoptosis, while high levels of P53 cells can be lead to apoptosis [15]. When HBx binds P53, the complex can block the nuclear P53, leading to lower concentrations of P53, which inhibits apoptosis. We found that Des increased expression of P53 in HCC cells. Thus, this may be an additional mechanism for Des' anti-cancer effect, which is different from the mechanism of annonaceous acetogenin-induced apoptosis [2].

In summary, our results show that Des' effect against HCC cells may be through upregulating expression of TLR4 and P53, which ultimately activates the innate immune response, allowing the immune system to clear cancer cells.
